# Utilizing large and diverse bacterial genome datasets to improve the detection and identification of Streptococcus pneumoniae via PCR-based diagnostics

**DOI:** 10.1099/mgen.0.001418

**Published:** 2025-06-09

**Authors:** Femke M. Ahlers, David J. Litt, Melissa J. Jansen van Rensburg, James E. Bray, Keith A. Jolley, Carmen Sheppard, Seyi Eletu, Juliana Coelho, Bruno Pichon, Odile B. Harrison, Martin C.J. Maiden, David W. Eyre, Norman K. Fry, Angela B. Brueggemann

**Affiliations:** 1 Nuffield Department of Population Health, University of Oxford, Oxford, UK; 2 Respiratory and Vaccine Preventable Bacteria Reference Unit, UK Health Security Agency, London, UK; 3 Department of Biology, University of Oxford, Oxford, UK; 4 Antimicrobial Resistance and Healthcare Associated Infection Reference Unit, UK Health Security Agency, London, UK; 5 Healthcare Associated Infections, Fungal, AMR, AMU & Sepsis Division, UK Health Security Agency, London, UK; 6 Immunisation and Vaccine Preventable Diseases, UK Health Security Agency, London, UK

**Keywords:** molecular diagnostics, non-pneumococcal *Streptococcus *species, qPCR, rapid diagnostic tests, *Streptococcus pneumoniae*, Xisco

## Abstract

The accurate identification of *Streptococcus pneumoniae* (pneumococcus) is crucial for diagnostics and surveillance but is complicated by the use of molecular assays that may also detect non-pneumococcal *Streptococcus* (NPS) species. Therefore, the aim of this study was to use a combination of *in silico* and *in vitro* analyses to evaluate PCR assays for the molecular detection and identification of pneumococci. A diverse dataset of over 9,300 pneumococcal and NPS genomes was investigated *in silico* to determine the sensitivity and specificity of assays for seven recommended gene targets: *lytA*, *piaB*, *ply*, *psaA*, Spn9802, SP2020 and Xisco. These *in silico* findings were used to design new diagnostic assays for two targets, Xisco and SP2020. The new assays were evaluated *in vitro* using three sets of isolates, one of which was selected based upon evidence for sequence diversity from a second *in silico* investigation of over 6,000 pneumococcal genomes sequenced by the United Kingdom Health Security Agency. Experimentally, the new Xisco and SP2020 assays were compared to published assays for *lytA* and *piaB*. The *in vitro* specificity was 100% (95% CI, 98.7–100%) across all assays. The *in vitro* sensitivity was 100% (95% CI, 98.5–100%) for *lytA*, SP2020_new and the Xisco assays and 99.6% (95% CI, 97.8–100%) for *piaB*. The new assays were found to be highly sensitive and specific and able to detect as few as two pneumococcal genome copies per quantitative PCR reaction. Overall, this study demonstrated the value of performing large-scale *in silico* genomic analyses of diagnostic targets, followed by *in vitro* testing that was specifically designed to account for global pneumococcal population-level diversity.

**Impact Statement**

The accuracy of a pneumococcal PCR-based diagnostic assay relies on targeting a gene that is ubiquitous in *Streptococcus pneumoniae* and absent from non-pneumococcal streptococci. The rapid expansion in publicly available genomic data over the past two decades provides an opportunity to exploit large and diverse bacterial genome datasets to design sensitive and specific PCR-based assays, which is demonstrated here. This study shows the potential of using *in silico* analyses followed by *in vitro* testing as a cost-effective way to test and develop molecular assays.

## Data Availability

All provenance and genome data are publicly available in PubMLST and can be accessed via the ID numbers in the Supplementary Data files. The pneumococcal data are available via the *Streptococcus pneumoniae* database (https://pubmlst.org/organisms/streptococcus-pneumoniae). The non-pneumococcal *Streptococcus* data are available via the Oral *Streptococcus* species database (https://pubmlst.org/organisms/oral-streptococcus-spp). The 248 pneumococci tested in the amplicon diversity panel were selected from the United Kingdom Health Security Agency (UKHSA) isolate collection. This collection was comprised of 2,434 stored isolates and genomes of surveillance pneumococci causing invasive disease in the UK between 2020 and 2022, plus 3,813 publicly available genomes [1,2], also available in the Pneumococcal Genome Library (https://pubmlst.org/organisms/streptococcus-pneumoniae/pgl). Five selected pneumococci (PubMLST ID 45894, 48094, 137601, 137353 and 137222) for the amplicon diversity panel could not be tested since the UKHSA freezer stocks were missing.

## Introduction

Worldwide, *Streptococcus pneumoniae* (pneumococcus) is a leading cause of severe invasive diseases such as pneumonia, meningitis and bloodstream infection, as well as often mild, self-limiting infections like otitis media and sinusitis. Pneumococci also colonize the healthy paediatric nasopharynx, which facilitates person-to-person transmission and is the first step in the pathway to disease [3]. Pneumococci are part of the Mitis group of streptococci, a subset of viridans streptococci that infrequently cause disease but are microbiologically similar and co-colonize the human nasopharynx and/or throat [4]. These related bacteria can complicate the accurate identification of pneumococci. The culture-based identification procedure for pneumococci recommended by the World Health Organization includes testing for optochin susceptibility and/or bile solubility to differentiate pneumococci from other related Mitis group species, but such testing may not always be performed, and anomalous results are possible for these phenotypic techniques [5]. As a consequence, the detection and reporting of other Mitis group streptococci instead of pneumococci can potentially lead to misdiagnoses, overestimations of carriage and antimicrobial resistance rates, or misinterpretations of vaccine impact [4,6, 7].

Molecular diagnostics such as PCR provide a rapid and accurate method to identify pneumococci and are particularly useful in clinical situations where urgent antimicrobial therapy is administered prior to sampling, resulting in nonviable bacteria in the specimen sent to the laboratory [6,8, 9]. The ideal amplification target for a pneumococcal PCR assay would be present in all pneumococci and absent from any other closely related bacterial species. Several genes have been recommended for use in such PCR assays, including *lytA* (autolysin), *piaB* (permease gene of the *pia* ABC transporter), *ply* (pneumolysin) and *psaA* (pneumococcal surface adhesin A), and recent genomic studies suggested alternative new targets including Spn9802 (putative transcriptional regulator), Xisco (putative surface-exposed protein) and SP2020 (putative GntR-family transcription regulator) [9,17]. However, concerns remain around whether PCR assays designed to amplify these recommended targets also detect sequences that are present in non-pneumococcal *Streptococcus* (NPS) species, or in prophages, which are ubiquitous amongst pneumococci and contain a *lytA* homologue [18,19].

The aim of this study was to use both *in silico* and *in vitro* methods to analyse current PCR targets in pneumococci and NPS species and assess the sensitivity and specificity of seven pneumococcal molecular assays. Additionally, the same approach was used to develop and test new PCR assays for two targets, Xisco and SP2020 [9,15]. In total, over 15,400 pneumococcal and NPS genome sequences, and three carefully chosen collections of bacterial isolates, were analysed.

## Methods

### Compilation of genome datasets used for *in silico* analyses

Overall, 9,372 publicly available genomes of 56 *Streptococcus* species were used for the initial *in silico* analyses. These included 7,547 diverse pneumococcal genomes from the PubMLST *S. pneumoniae* isolate collection and a subset of the curated Pneumococcal Genome Library (https://pubmlst.org/organisms/streptococcus-pneumoniae/pgl), which is an open-access compilation of genomes and metadata from published papers (Data S1, available in the online Supplementary Material) [20]. In addition, 1,825 genomes of 55 NPS species from the public Oral *Streptococcus* species database (https://pubmlst.org/organisms/oral-streptococcus-spp) were analysed (Data S2) [20,21].

The NPS dataset included genomes of *Streptococcus* species that cause human disease, colonize the oral cavity and/or are genetically closely related to pneumococcus. The ribosomal MLST (rMLST) scheme characterizes genetic variation within the 53 bacterial ribosomal proteins, and rMLST is especially useful for predicting the bacterial species [21]. Quality control metrics were applied as follows: (i) check that the bacterial species assigned by the submitting laboratory matched the rMLST species identification; (ii) number of contigs ≤500; (iii) N50 ≥20,000 bp; (iv) only one rMLST allele per non-paralogous locus; (v) complete rMLST allelic profile (i.e. 53 ribosomal loci). Amongst genomes that passed all five criteria, one genome per unique ribosomal sequence type (rST) was chosen. The dataset was compiled in March 2021, and a maximum of 180 genomes of any one *Streptococcus* species was included, to achieve a reasonable distribution of genomes across all species based on the number of genomes available at the time. If more than 180 genomes were available, then neighbour-joining trees were constructed via PubMLST based upon the 53 concatenated rMLST sequences and visualized in Interactive Tree of Life [22]. Obvious outliers were removed, and a maximum of 180 genomes with different rSTs distributed across the tree were chosen per species. rSTs present more frequently in the database, and those genomes with the lowest number of contigs were prioritized in the final selection, where necessary, to make a final decision.

### *In silico* assessment of PCR primers and probes

Three new assays targeting Xisco and new primers for SP2020 were designed using Geneious Prime and Primer Express 3 (Thermo Fisher Scientific) and the Xisco and SP2020 sequence alignments from the pneumococcal *in silico* dataset. The *in silico* performance of these assays was compared to seven existing PCR assays (targeting *lytA*, *piaB*, *psaA*, *ply*, Spn9802, SP2020 and Xisco; see Table S1 for details and primer/probe sequences). Analyses were performed using the In-silico PCR Experiment Simulation System (ipcress) software built into BIGSdb, which deduced whether forward and reverse PCR primer sequences would theoretically result in an amplicon, given the input bacterial sequence [23,24]. Conservatively, up to four nucleotide mismatches per primer binding region were allowed, and the maximum predicted amplicon length was set to three times the predicted amplicon length in pneumococcal reference genome TIGR4 (Table S1). Next, the predicted PCR amplicon sequences were assessed using Geneious Prime Software, whereby unique amplicon sequences (i.e. sequence variants) were selected and aligned using the muscle algorithm with eight iterations [25,26]. Probe sequences were then added to the PCR amplicon sequence alignment to predict whether the probe would anneal to the predicted amplicon, allowing for up to four nucleotide mismatches per probe/amplicon.

In order to assess the accuracy of each set of primers/probes, Youden’s J statistic was calculated [27]:


$$Youden’sJstatistic=\frac{Truepositivepneumococcalgenomes}{Totalpneumococcalstudydataset}+\frac{TruenegativeNPSgenomes}{TotalNPSdataset}-1$$

### Selection of pneumococci and NPS isolates for *in vitro* testing

Three sets of isolates were chosen for *in vitro* testing of the PCR assays: (i) pneumococci received in April 2022 as part of the United Kingdom Health Security Agency (UKHSA) invasive disease surveillance of isolates from patients in England (‘sensitivity panel’), (ii) NPS isolates from UKHSA collections (‘specificity panel’) and (iii) pneumococci selected from UKHSA collections based upon their predicted PCR amplicon sequences (‘amplicon diversity panel’).

Pneumococci considered for inclusion in the amplicon diversity panel had already been whole-genome sequenced prior to this study, and the genomes met these quality control criteria: (i) <500 contigs; (ii) total genome length, 1.9–2.3 Mb; (iii) complete MLST allelic profile (seven loci); and (iv) complete rMLST allelic profile (53 loci). Assays targeting *piaB*, *lytA*, SP2020_new and the three Xisco assays were assessed *in silico* (as described above), and pneumococci with unique nucleotide sequences for each amplicon were selected for *in vitro* testing [10,12]. When there were multiple pneumococci with the same *in silico* PCR amplicon sequence, isolates were chosen to optimize serotype diversity, and genomes with the lowest number of contigs were prioritized. In addition, representative pneumococci that were negative for any *in silico* PCR amplicons were chosen for *in vitro* testing if (i) the target gene was absent, (ii) there were up to four nucleotide mismatches in the primer binding region and a deletion or >4 nucleotide mismatches in the probe binding region and (iii) there was a contig break within the target gene.

### Preparation of DNA extracts for *in vitro* testing

Freezer stocks of streptococcal isolates were cultured on Columbia blood agar plates and incubated overnight at 36.5 °C plus 5% CO_2_. DNA was extracted from the plated growth using the QIAsymphony DSP DNA Mini Kit on a QIAsymphony SP automated extractor (QIAGEN), following the manufacturer’s protocol for extraction of DNA from bacteria, including (a) a 1 h pre-lysis step at 56 °C in lysis buffer ATL (QIAGEN) with proteinase K and RNAse A treatment for pneumococci or (b) a 1 h pre-lysis step at 37 °C in resuspension buffer P1 (QIAGEN) with mutanolysin, lysozyme and RNAse A followed by a 2 h incubation at 56 °C after the addition of proteinase K for NPS [28,29].

### Genome sequencing

Bacterial DNA was sequenced on the Illumina NextSeq 1000 platform and assembled as previously described [1]. All isolate metadata and corresponding genome assemblies were uploaded to PubMLST for further analyses.

### Standardization of DNA concentrations

DNA was quantified with the Qubit dsDNA BR Assay Kit on a Qubit 4 Fluorometer (Invitrogen), following the assay kit protocol. DNA extracts were standardized to 0.1 ng µl^−1^ in TE buffer (Tris-HCl 10 mM (pH 8.0), EDTA 1 mM) for testing. DNA extracts tested in the *in vitro* sensitivity and specificity panels were diluted 1 : 100 in TE buffer.

### Design of quantitative PCR probes

Two probes with different fluorophores were utilized in SP2020 assays: (i) a FAM-labelled probe for limit of detection experiments against the FAM-labelled Xisco assays and (ii) a CY3-labelled probe for multiplexing the assay. A quantitative PCR (qPCR) assay targeting universal 16S rDNA as a positive control for the presence of bacterial DNA was used as previously published, except that a CY5 fluorophore was added to allow multiplexing [30].

### Testing of multiplex qPCR assays

*In vitro* testing followed the Minimum Information for Publication of Quantitative Real-Time PCR Experiments guidelines [31]. The Xisco, SP2020_new and 16S rDNA primers and probes were multiplexed in a 20 µl reaction mixture that contained 1X TaqPath qPCR Master Mix (Applied Biosystems), Xisco_1 or Xisco_2 or Xisco_3 primers and FAM-labelled probe, SP2020 primers and CY3-labelled probe and 16S rDNA primers at 0.3 µM, 16S rDNA CY5-labelled probe at 0.1 µM and 2 µl target DNA.

For comparison, *lytA*, *piaB* and 16S rDNA were multiplexed in a 20 µl reaction mixture that contained 1X TaqMan Fast Advanced Master Mix (Applied Biosystems), *lytA* primers and FAM-labelled probe and *piaB* primers and JOE-labelled probe at 0.2 µM, 16S rDNA primers at 0.3 µM, 16S rDNA CY5-labelled probe at 0.1 µM and 2 µl target DNA. Testing was performed in 96-well plates that included pneumococcal DNA and molecular-grade water as the positive and negative controls, respectively. DNA was amplified in the QuantStudio 5 system (Applied Biosystems) using the following cycling conditions: 50 °C for 2 min, 95 °C for 20 s, followed by 40 cycles of 95 °C for 3 s and 60 °C for 30 s.

### Determining the limit of detection of the newly designed assays

A pneumococcal DNA extract, for which the genome sequence had no nucleotide mismatches in the primer or probe binding regions of Xisco or SP2020, was used to determine the limit of detection for each qPCR assay. The average genome length (2,115,978 bp) was calculated using 38 complete genomes from the pneumococcal *in silico* study dataset. This value was used to convert the weight of DNA (fg) in each sample of three independent dilution series to an estimated number of pneumococcal genome copies. To compare fairly the limit of detection of singleplex assays, FAM-labelled probes were used for all assays. For Xisco_1, Xisco_2, Xisco_3 and SP2020_new, the 20 µl reaction mixture contained 1X TaqPath qPCR Master Mix (Applied Biosystems), Xisco_1 or Xisco_2 or Xisco_3 or SP2020 primers and FAM-labelled probe at 0.3 µM and 2 µl target DNA. The limit of detection was measured for the multiplex assays, using the same qPCR protocol described previously when testing the *in vitro* panels.

*In vitro* data were analysed using the Applied Biosystems Design and Analysis Software 2.6.0 (Thermo Fisher Scientific). Figures and exact 95% CIs were generated using R version 4.2.1. The efficiency of the singleplex assays was calculated as follows:


$$Efficiency=10^{-\frac{1}{slope}}-1$$

where the slope was determined by fitting a linear model of C_q_ (quantification cycles) value vs. pneumococcal DNA copy number per well.

## Results

### *In silico* analyses of pneumococcal diagnostic targets

**Fig. 1.**
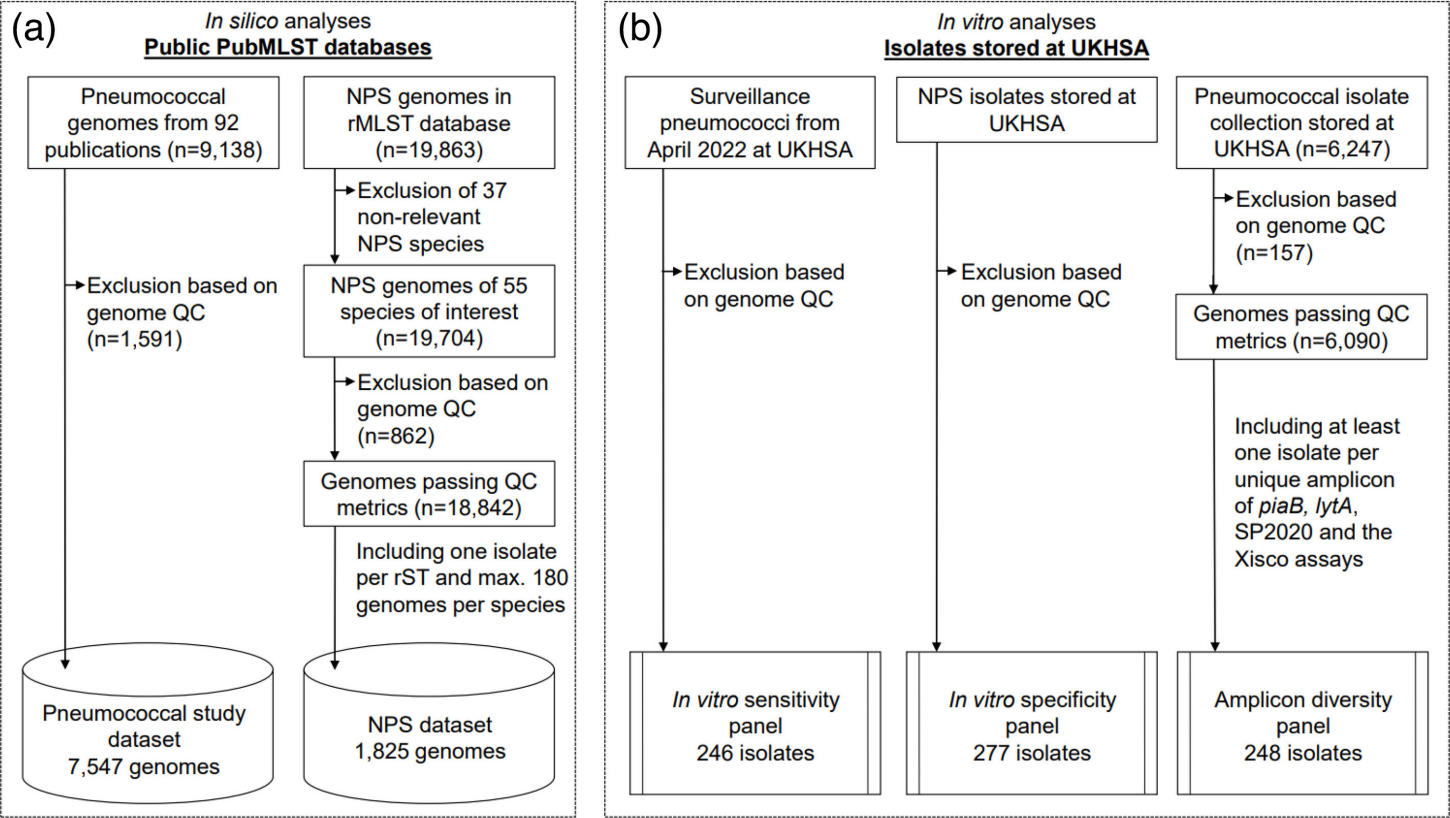
Schematic overview of the genome datasets and bacterial isolates analysed in this study. (**a**) Description of the two genome datasets used for *in silico* analyses. (**b**) Pneumococcal and NPS isolates tested in the *in vitro* analyses. Note: QC, quality control.

The pneumococcal genome dataset (*n*=7,547; Figs 1a, S1 and S2 and Data S1) contained a diverse set of carriage and invasive pneumococci collected between 1916 and 2018 from 43 different countries across six different continents. Ninety-four different serotypes and 1,036 MLST sequence types (STs) were represented.

**Fig. 2.**
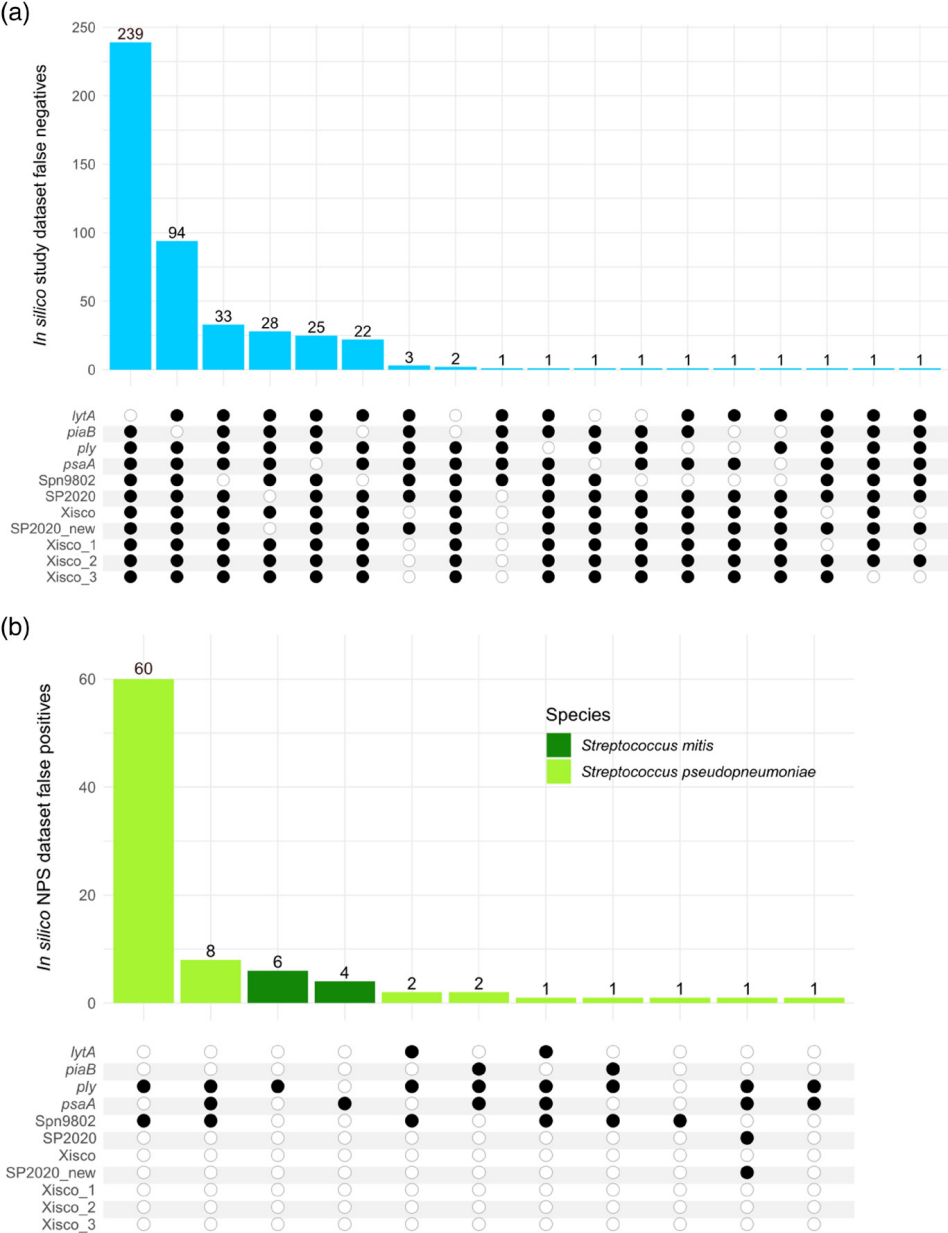
Summary of pneumococcal and NPS genomes causing discrepancies after *in silico* assessments of pneumococcal PCR assays. Black circles indicate that a PCR amplicon was detected, and white circles indicate *in silico* negative tests. (**a**) Pneumococcal genomes that were *in silico* negative (*n*=456) for one or more PCR assays are shown, stratified by combinations of *in silico* positive tests. (**b**) NPS genomes that were *in silico* positive (*n*=87) for one or more pneumococcal PCR assays, stratified by combinations of positive tests.

The pneumococcal genomes were analysed *in silico* to assess the suitability of primers and probes designed for use in seven previously published PCR-based assays and four new assays (Tables 1 and S1). All seven previously published assays resulted in an *in silico* positivity rate >96% (range, 96.8–100%). Most genomes had only one *in silico* PCR amplicon per assay; however, multiple PCR amplicons were identified for *piaB* and Spn8902 (up to two each), *lytA* (up to 4), *ply* and SP2020 (up to three each). Between 3 and 243 genomes were *in silico* negative for one or more of these seven assays (Table 1, Fig. 2a). The *lytA* assay had the greatest number of negative results (*n*=243), although this could be overestimated due to genome assembly issues related to the concomitant presence of prophage *lytA* homologues.

**Table 1.** Summary of the *in silico* assessment of published and newly designed primers and probes within the pneumococcal genome dataset (*n*=7,547)

Target	Genomes with x amplicons (*n*)					Unique amplicons (*n*)	Range of amplicon length (bp)	Predicted amplicon positive (%) (CI_95%_)
	x=**0**	x=**1**	x=**2**	x=**3**	x=**4**			
lytA*	243	6,769	469	57	9	17	74–75	96.8 (96.4–97.2)
piaB*	120	7,426	1	0	0	19	104	98.4 (98.1–98.7)
ply*	3	7,523	15	6	0	13	78	100 (99.9–100)
psaA*	27	7,520	0	0	0	18	113–115	99.6 (99.5–99.8)
Spn9802*	59	7,483	5	0	0	30	154–158	99.2 (99.0–99.4)
SP2020*	29	7,512	5	1	0	13	155	99.6 (99.4–99.7)
Xisco*	6	7,541	0	0	0	159	431–581	99.9 (99.8–100)
SP2020_new†	29	7,512	5	1	0	13	143	99.6 (99.4–99.7)
Xisco_1†	6	7,541	0	0	0	41	123–153	99.9 (99.8–100)
Xisco_2†	4	7,543	0	0	0	38	101–125	99.9 (99.9–100)
Xisco_3†	6	7,541	0	0	0	38	159–255	99.9 (99.8–100)

*Previously published PCR assay (see Table S1).

†Newly designed qPCR primers and probes (see Table S1).

Amongst the previously published assays, the number of unique *in silico* PCR amplicons ranged from 13 to 159, and sequence alignments of the unique amplicons revealed several instances of nucleotide mismatches in primer and probe binding regions (Table 1, Figs S3 and S4). Xisco had the greatest sequence diversity (159 unique amplicons), whereas *ply* and SP2020 sequences were the most conserved (13 unique amplicons each).

The newly designed primers and probes for SP2020 (SP2020_new) and Xisco (three versions; Table 1) had an overall *in silico* positivity rate >99% (range, 99.6–99.9%) and generally a smaller number (4–29) of genomes with no *in silico* amplicon. Only the SP2020_new primers resulted in multiple *in silico* amplicons (up to three amplicons, but only involving six genomes in total), and there were between 13 and 41 unique amplicons amongst the newly designed targets (Figs S5–S7).

The pneumococcal genomes causing discrepancies *in silico* were inspected in detail (Fig. 2a). Overall, 94% (7,091 out of 7,547) of the pneumococcal genomes were *in silico* positive for all of the pneumococcal assays listed in Table 1; however, amongst those 456 pneumococcal genomes that were *in silico* negative for one or more of the assays, over half (*n*=239) were only negative for *lytA*, and another 21% (*n*=94) were only negative for *piaB* (Fig. 2a).

### *In silico* analyses of pneumococcal targets within NPS genomes

The NPS dataset contained 1,825 genomes of 55 NPS species (Figs 1a and S8 and Data S2). The number of genomes per NPS species ranged from 1 to 180 (180 was the self-imposed upper limit based on available genomes). *Streptococcus agalactiae*, *Streptococcus pyogenes*, *Streptococcus suis*, *Streptococcus mutans* and *Streptococcus dysgalactiae* were most frequently represented in the dataset (153–180 genomes per species) and a further 25 NPS species were represented by 5–102 genomes each (Fig. S8).

NPS genomes were analysed *in silico* using the same pneumococcal assays, and >95% (range, 95.5–100%) of the NPS genomes were negative for each of the pneumococcal assays (Table 2). Xisco was the only pneumococcal target with no detectable *in silico* amplicons amongst NPS genomes.

**Table 2.** Summary of the *in silico* assessment of published and new primers and probes within the NPS genome dataset (*n*=1,825)

Target	Genomes with x amplicons (*n*)			Predicted amplicon negative (%) (CI_95%_)
	x=**0**	x=**1**	x=**2**	
lytA*	1,822	3	0	99.8 (99.5–100)
piaB*	1,822	3	0	99.8 (99.5–100)
ply*	1,743	79	3	95.5 (94.5–96.4)
psaA*	1,808	17	0	99.1 (98.5–99.5)
Spn9802*	1,752	73	0	96.0 (95.0–96.9)
SP2020*	1,824	1	0	99.9 (99.7–100)
Xisco*	1,825	0	0	100 (99.8–100)
SP2020_new†	1,824	1	0	99.9 (99.7–100)
Xisco_1†	1,825	0	0	100 (99.8–100)
Xisco_2†	1,825	0	0	100 (99.8–100)
Xisco_3†	1,825	0	0	100 (99.8–100)

*Previously published PCR assay (Table S1).

†Newly designed qPCR primers and probes (Table S1).

Over 95% (1,738 out of 1,825) of the NPS genomes were *in silico* negative for all pneumococcal targets; however, 87 *Streptococcus pseudopneumoniae* and *Streptococcus mitis* genomes were *in silico* positive for up to four pneumococcal targets (Fig. 2b). All 77 *S*. *pseudopneumoniae* genomes in the NPS dataset were *in silico* positive for one or more pneumococcal targets apart from Xisco. The most common combination was *ply* and Spn9802, detected amongst 60 *S*. *pseudopneumoniae* genomes, but only one *S. pseudopneumoniae* genome was positive for SP2020_new. Ten out of 84 *S*. *mitis* genomes were *in silico* positive for *ply* or *psaA* (Fig. 2b).

Youden’s J statistic was calculated to assess the overall accuracy of the previously published assays based upon these *in silico* data. Xisco and SP2020 had the highest Youden’s J value (1.00), followed by *psaA* (0.99), *piaB* (0.98), *lytA* (0.97), *ply* and Spn9802 (0.95), indicating that Xisco and SP2020 were the most accurate targets. This analysis, plus the Xisco sequence diversity and variation in amplicon length (431–581 bp) using the previously published Xisco PCR primers, motivated the design of Xisco qPCR primers and probes to further increase assay sensitivity and specificity. In addition, a new set of primers was designed for SP2020, for use in a multiplex assay with Xisco, since the existing SP2020 qPCR primers did not have a compatible melting temperature (Table S1 and Figs 5–7).

### Limit of detection experiments

**Fig. 3.**
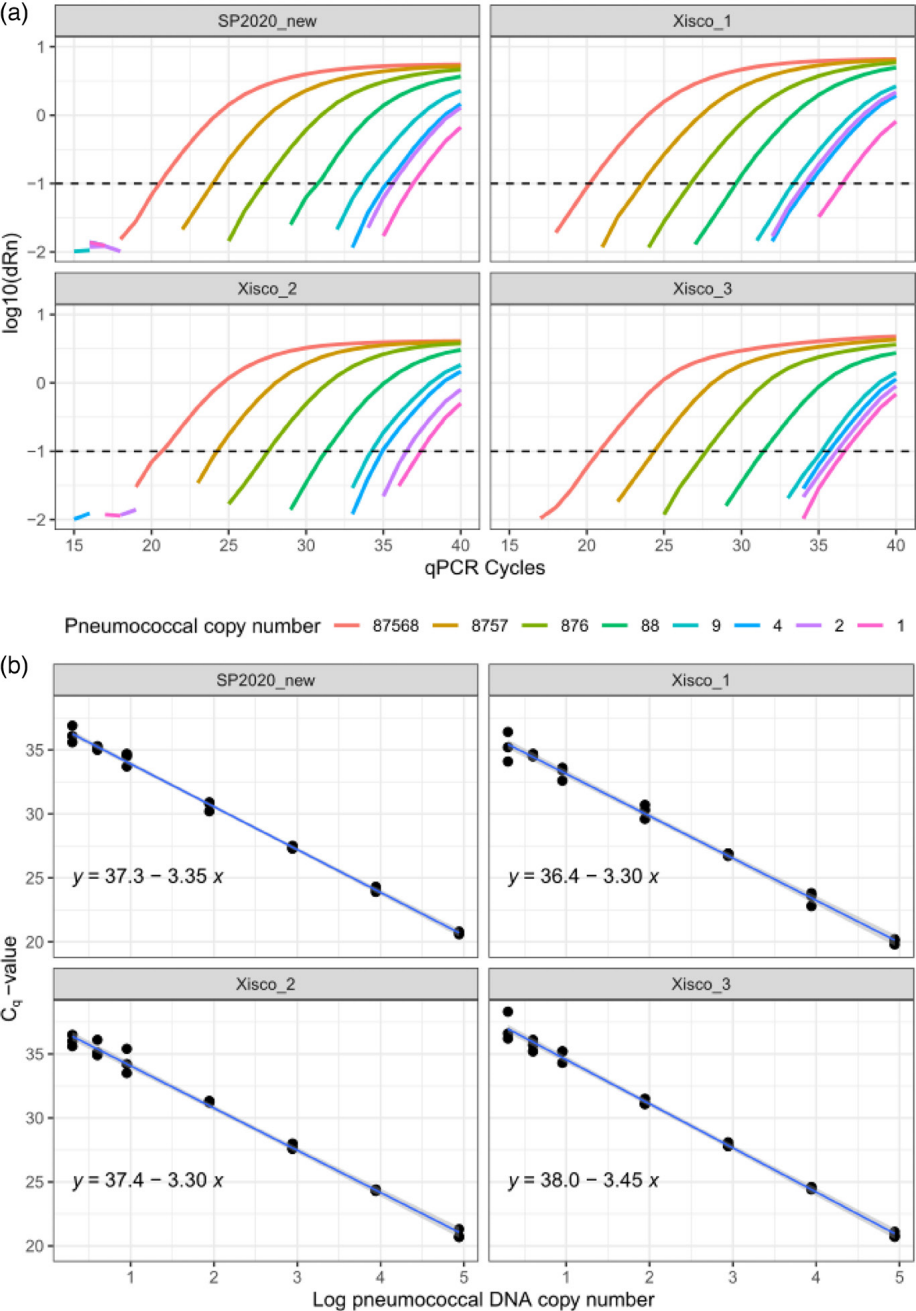
Limit of detection of Xisco_1, Xisco_2, Xisco_3 and SP2020_new singleplex assays. (**a**) Representative results for one of the three dilution series, depicting qPCR curves coloured by pneumococcal copy number per well. Horizontal dashed lines indicate the fluorescence threshold at 0.1 dRn. (Note that non-specific amplification background noise is present in the two left panels between 15 and 20 qPCR cycles and should be ignored.) (**b**) A linear model fitted to the results for all three dilution series (24 data points in total), with 95% CIs in grey.

Three independent serial dilutions of extracted pneumococcal DNA were used to determine the limit of detection of the new Xisco (three versions) and SP2020_new assays (Fig. 3a). All singleplex assays consistently detected a minimum of two pneumococcal DNA copies per reaction, and the qPCR efficiency was >0.95 (Fig. 3b).

Assays were multiplexed in several combinations: Xisco_1 (or Xisco_2 or Xisco_3), SP2020_new and 16S rDNA and *lytA*, *piaB* and 16S rDNA. Each multiplex assay consistently detected a minimum of nine pneumococcal genome copies per well at a C_q_-value below 37, and the results of the limit of detection experiments were used to determine fluorescence thresholds (Tables S2–S5). These thresholds were applied to subsequent *in vitro* testing as described below.

### Testing the *in vitro* sensitivity and specificity of new qPCR assays

A total of 246 invasive pneumococci recovered in 2022 were used to test assay sensitivity (Fig. 1b and Data S3). The pneumococci represented 75 STs, 122 rSTs and 25 serotypes, and the five highest frequency serotypes were 8 (*n*=42), 3 and 22F (*n*=29 each), 19A (*n*=21) and 9N (*n*=13). All 246 pneumococci were *in vitro* positive for *lytA*, SP2020_new and Xisco (sensitivity, 100%, 95% CI, 98.5–100%). One pneumococcal isolate was *in vitro* negative for *piaB* (sensitivity, 99.6%, 95% CI, 97.8–100%). *In silico* analyses confirmed the *in vitro* results.

A total of 277 isolates of 21 different NPS species were tested to determine assay specificity: *S. mutans* (*n*=31), *Streptococcus intermedius* (*n*=29), *Streptococcus parasanguinis* (*n*=26), *Streptococcus lutetiensis* (*n*=24), *Streptococcus gallolyticus* and *Streptococcus sanguinis* (*n*=23 each), *Streptococcus gordonii* and *Streptococcus pasteurianus* (*n*=21 each), *S. mitis* (*n*=20), *Streptococcus oralis* (*n*=17), *S. pseudopneumoniae* (*n*=11), *Streptococcus constellatus* (*n*=8), *Streptococcus sobrinus* (*n*=7), *Streptococcus infantarius* (*n*=6), *Streptococcus cristatus* (*n*=3), *Streptococcus downei* (*n*=2), *Streptococcus massiliensis*, *Streptococcus minor*, *Streptococcus peroris*, *Streptococcus pseudoporcinus* and *Streptococcus salivarius* (*n*=1 each; Data S4). Overall, 257 unique rSTs were represented. All isolates were *in vitro* negative for *lytA*, *piaB*, SP2020_new and the Xisco assays (specificity, 100%, 95% CI, 98.7–100%). *In silico* analyses were performed to confirm the *in vitro* results, and one discrepancy was found: the ipcress tool predicted one *lytA* positive *S. mitis*, but the identification of nucleotide mismatches in the primers and probe explained the *in vitro* negative result.

### *In vitro* testing of pneumococci that were selected based upon *in silico* analyses

A dataset of 6,090 UKHSA pneumococcal genomes from UK bacterial isolates that were available for *in vitro* experiments was analysed *in silico* (as described above) for *lytA*, *piaB*, SP2020_new and Xisco (three versions) primers and probes (Fig. 1b and Table S6). The *in silico* results were similar to those of the 7,547 genomes from PubMLST; therefore, 248 UKHSA pneumococci were chosen for *in vitro* testing based upon their *in silico* amplicon sequences (Table 3 and Data S5). The *in silico* and *in vitro* results were concordant for all pneumococci that were *in silico* positive for the tested assays. Amongst pneumococci that were negative *in silico* but positive *in vitro*, these discordant results were mainly due to missing genomic sequence data (e.g. incomplete target sequence at the end of a contig).

**Table 3.** Summary of *in silico* and *in vitro* test results for UKHSA pneumococci of the amplicon diversity panel (*n*=248)

Target	Genomes with x *in silico* amplicons (*n*)			Unique amplicons (*n*)	*In silico* amplicons (total *n*)	*In vitro* positive (*n*)	*In silico* negative		
	x=**0**	x=**1**	x=**2**				Deletion in probe binding region (*n*)	End of contig (*n*)	No amplicon (*n*)
lytA*	31	197	20	11	217	247	0	25	6
piaB*	22	226	0	14	226	228	0	0	22
SP2020_new†	12	236	0	9	236	239	0	3	9
Xisco_1†	0	248	0	42	248	248	--	--	--
Xisco_2†	0	248	0	31	248	248	--	--	--
Xisco_3†	1	247	0	43	247	247	1	0	0

*Previously published PCR assay (see Table S1).

†Newly designed qPCR primers and probes (see Table S1).

**Fig. 4.**
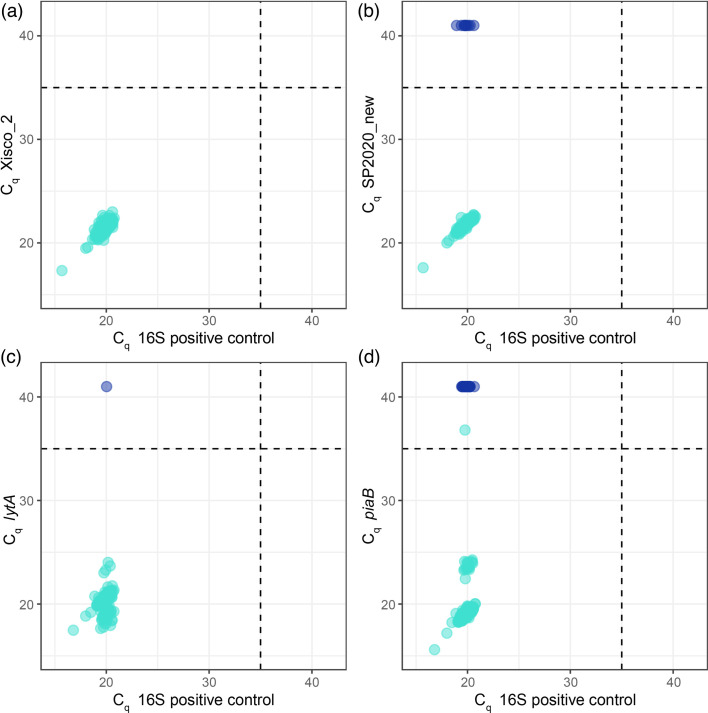
Relationship between experimental C_q_-values for the 16S rDNA positive control and each of the four targets. Multiplex assays were used to test 248 UKHSA pneumococcal isolates selected based upon *in silico* amplicon diversity. Dark blue circles are placeholders for negative SP2020_new, *lytA* and *piaB* assays, in panels (b), (c) and (d), respectively. Note: data for Xisco_1, _2 and _3 were very similar, so only Xisco_2 is shown here.

The three Xisco *in vitro* assays had the highest number of unique amplicons (range 31–43), compared to 9 unique amplicon sequences for SP2020_new, 11 for *lytA* and 14 for *piaB* (Table 3, Fig. 4). Inclusion of the 16S rDNA PCR target in multiplex assays was a positive control and allowed comparison of the C_q_-values of the different qPCR targets (*lytA*, *piaB*, SP2020_new and three Xisco assays). Overall, despite some sequence variability across many Xisco amplicons, the C_q_-values of the Xisco PCR reaction against the C_q_-values of the 16S rDNA positive control PCR clustered well together, demonstrating that the Xisco assays performed well across a range of amplicon sequences (Fig. 4).

## Discussion

For over three decades, molecular diagnostics have been successfully employed in clinical microbiology and research laboratories to identify, characterize and quantify a wide range of microbial pathogens. In recent years, the number of publicly available whole-genome sequences has increased rapidly, leading to a shift in our understanding of infectious diseases and the microbes that cause them, but also creating opportunities to re-use these genomic data for analyses beyond the original research question and thus maximize the benefits of the investment in genome sequencing [32,35]. An earlier study of 1,700 *Campylobacter* species genomes used a similar approach to our current study, i.e. the authors used *in silico* analyses of the genome sequences to design a real-time PCR assay that targeted two genes and successfully differentiated *Campylobacter jejuni* and *Campylobacter coli* with high test sensitivity and specificity [35]. We exploited the volume of publicly available bacterial genome data to perform large-scale *in silico* genomic analyses to assess published diagnostic qPCR primers and probes recommended for use in the detection of pneumococci. Those *in silico* results were used to further develop and test qPCR *in vitro* assays.

The published assays all proved to be highly sensitive and specific, but specificity was further improved by developing new assays for Xisco and SP2020. This avoided misidentifying closely related streptococci like *S. mitis* and *S. pseudopneumoniae*, which can be problematic to differentiate from pneumococci, especially in nasopharyngeal samples where co-colonization is frequent. The newly designed qPCR assays were highly accurate and detected low copy numbers of pneumococcal DNA, making the assays useful for samples with a low bacterial yield. They are promising candidates as improved diagnostic PCR assays to detect *S. pneumoniae.*

A limitation of this study is that we did not assess every published pneumococcal molecular diagnostic assay available but instead chose one assay for each of the seven genetic targets. We also performed the *in vitro* testing with DNA extracted from pure bacterial cultures, and it would be useful in future work to assess how well these new qPCR assays perform when used as diagnostic assays directly on clinical specimens to avoid the need for bacterial culture and/or to detect pneumococci in clinical samples when the pathogen is nonviable.

The strengths of this study were the inclusion of a large number of pneumococcal genomes to assess sequence diversity at different genetic targets, and also the use of a large collection of NPS genomes across a wide range of *Streptococcus* species to evaluate assay specificity. The bioinformatics tools to assess PCR primers *in silico* are freely available and easy to use, and the *in silico* data generated can be deployed to inform targeted selection of representative bacterial isolates for *in vitro* testing. This reduces the overall cost and time required for testing whilst maximizing the sequence diversity of the tested isolates [35]. Altogether, this *in silico* plus *in vitro* approach provided a rapid and cost-effective way to assess and improve pneumococcal molecular diagnostics, and the methodology could be applied to any organism for which a reasonable number of genomes are available for analysis.

## Supplementary material

10.1099/mgen.0.001418 Uncited Supplementary Material 1.

10.1099/mgen.0.001418 Uncited Supplementary Material 2.

10.1099/mgen.0.001418 Uncited Supplementary Material 3.

## Abbreviations

ipcress: In-silico PCR Experiment Simulation System
NPS: non-pneumococcal *Streptococcus*
NPS: non-pneumococcal *Streptococcus*
qPCR: quantitative PCR
rMLST: ribosomal MLST
rST: ribosomal sequence type
STs: sequence types
UKHSA: United Kingdom Health Security Agency
