# Supplementary material for: Utilizing large and diverse bacterial genome datasets to improve the detection and identification of Streptococcus pneumoniae via PCR-based diagnostics
Source: Microb Genom. 2025 Jun 9;11(6):001418. doi: 10.1099/mgen.0.001418 (PMC12149409; doi:10.1099/mgen.0.001418)
Supplement: Uncited Supplementary Material 1. [file mgen-11-01418-s001.pdf]

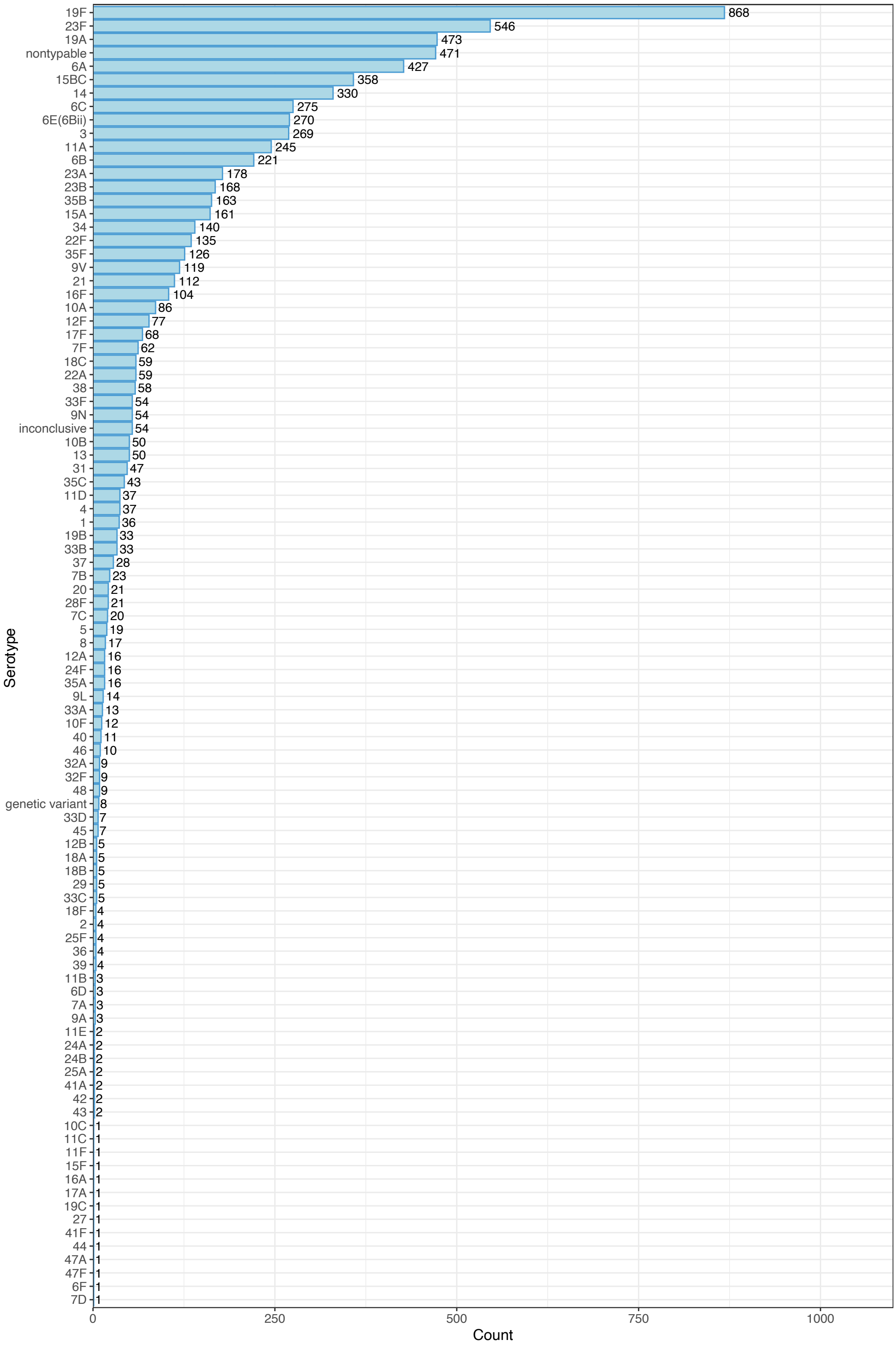

Supplementary Figure 1. Distribution of serotypes for the *in silico* pneumococcal study dataset (n=7,547).

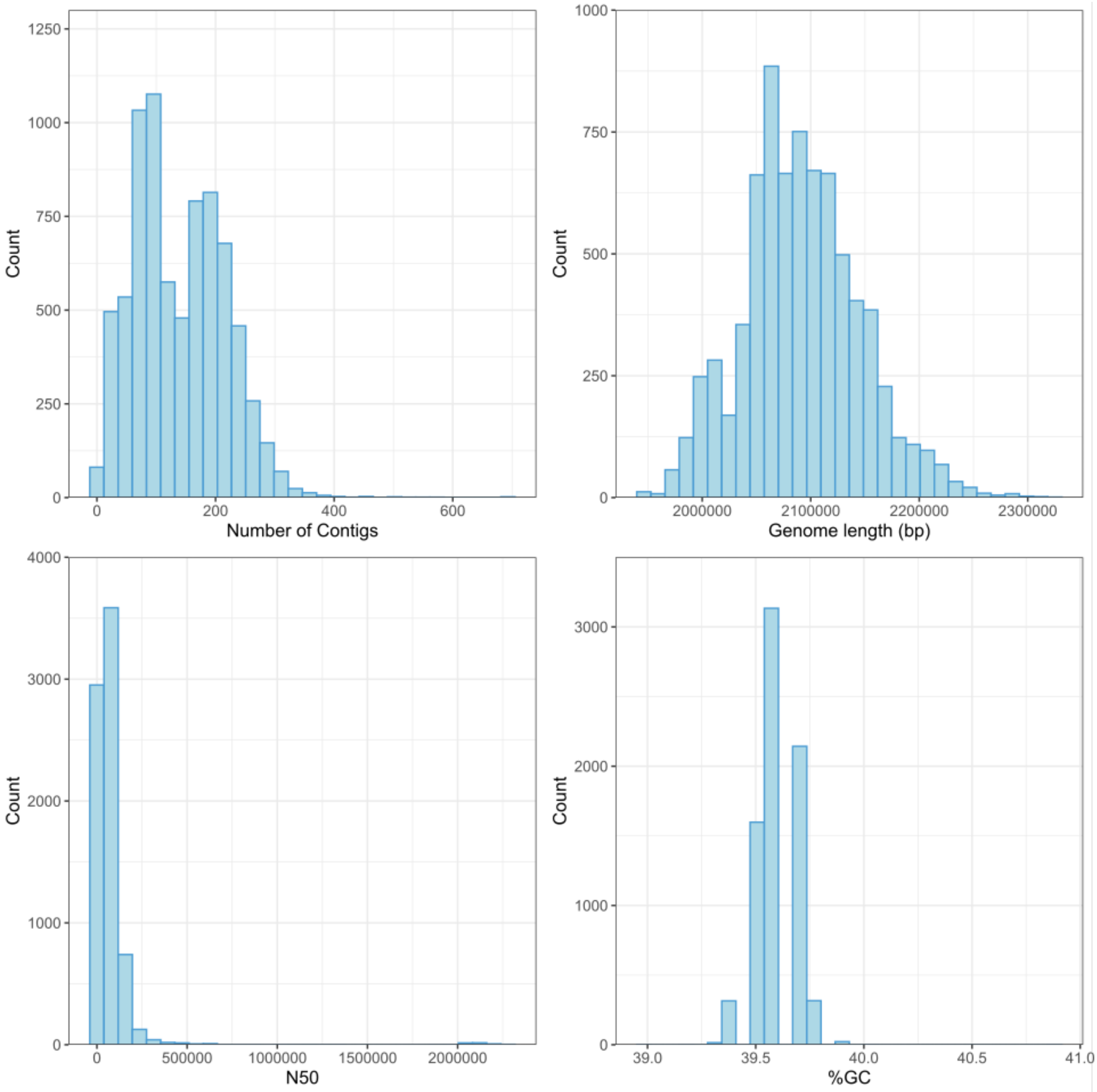

Supplementary Figure 2. Genome quality data for the *in silico* pneumococcal study dataset (n=7,547).

Unique *lytA in silico* amplicons

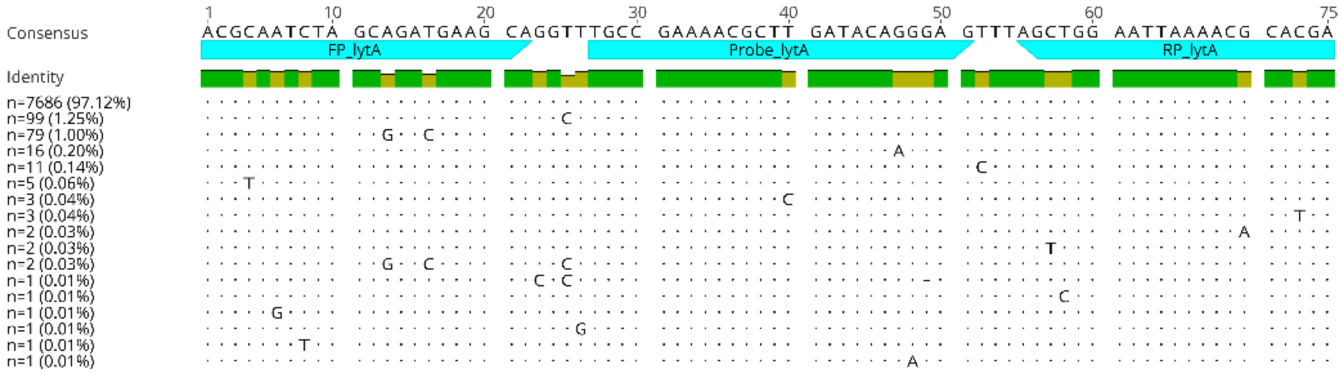

Unique *piaB in silico* amplicons

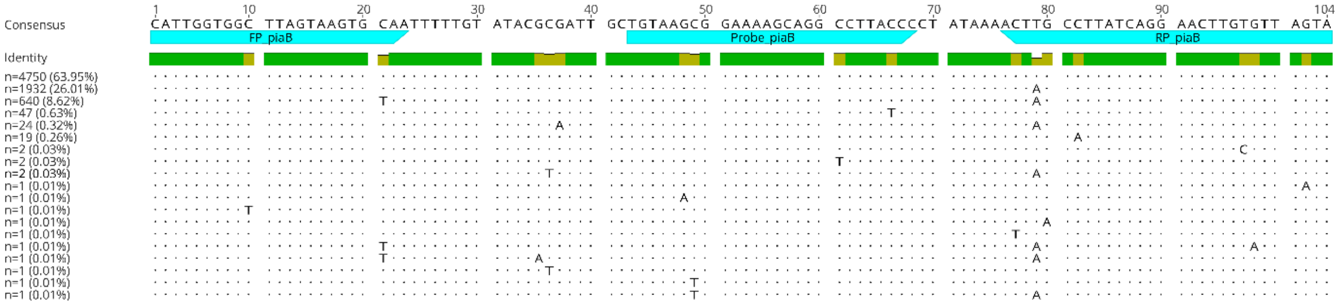

Unique *ply in silico* amplicons

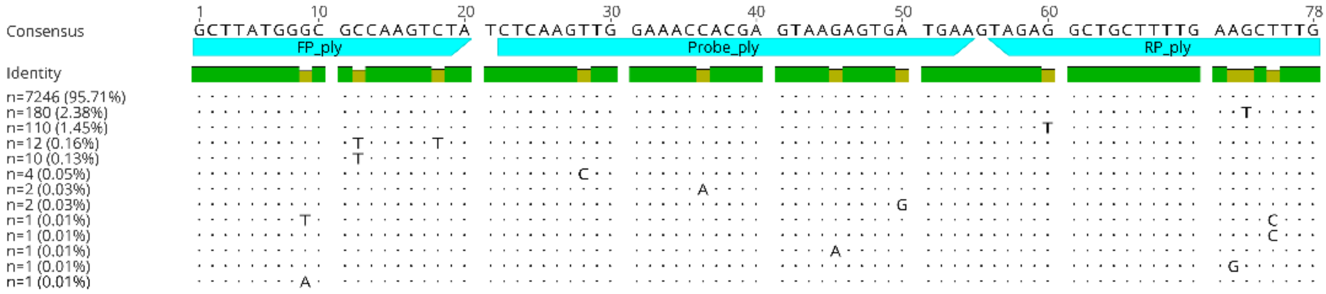

Unique *psaA in silico* amplicons

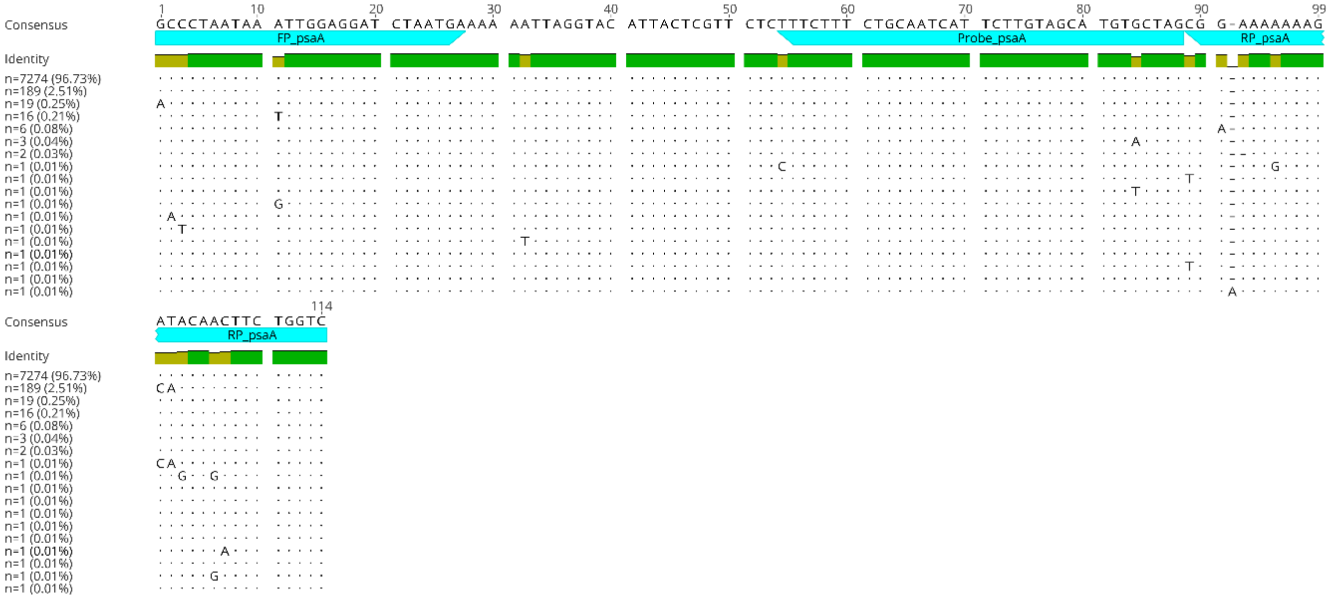

Supplementary Figure 3. Nucleotide sequence alignments of *in silico* PCR amplicons for four previously published targets. The consensus sequence was determined based upon the nucleotide in each position that represents at least 50% of nucleotides in that position, across the set of sequences being compared. Primer and probe binding regions are annotated (FP, forward primer; RP, reverse primer). The 'identity' row depicts nucleotide sequence pairwise identity at each position: green, 100%; brown, 30-100%. In the first column, n (%) indicates the frequency of that amplicon sequence out of the total number of predicted amplicons in the *in silico* pneumococcal study dataset for that assay. Dots indicate that the nucleotide is identical to the consensus; dashes indicate a sequence gap.

Unique SP2020 *in silico* amplicons

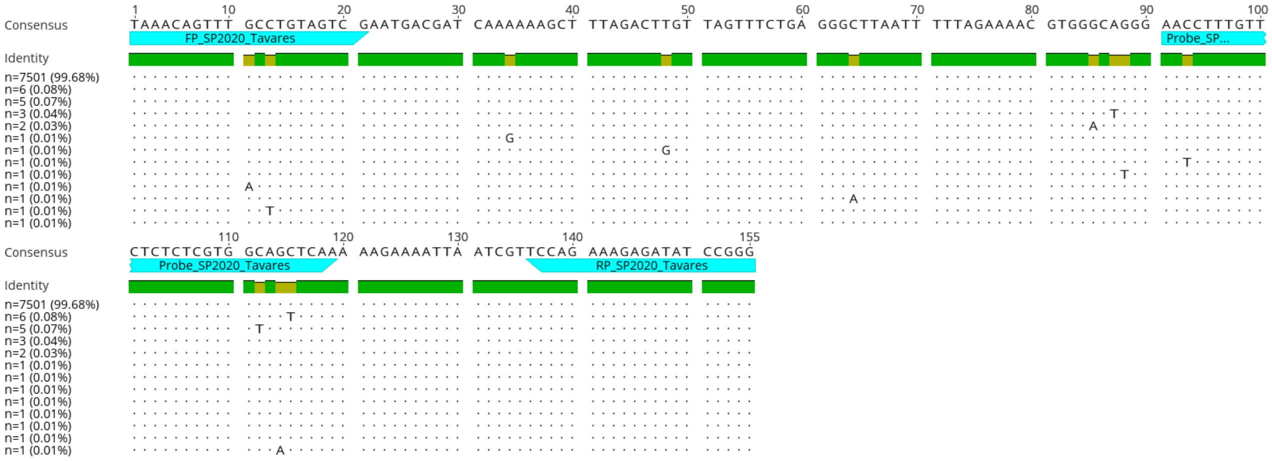

Unique Spn9802 *in silico* amplicons

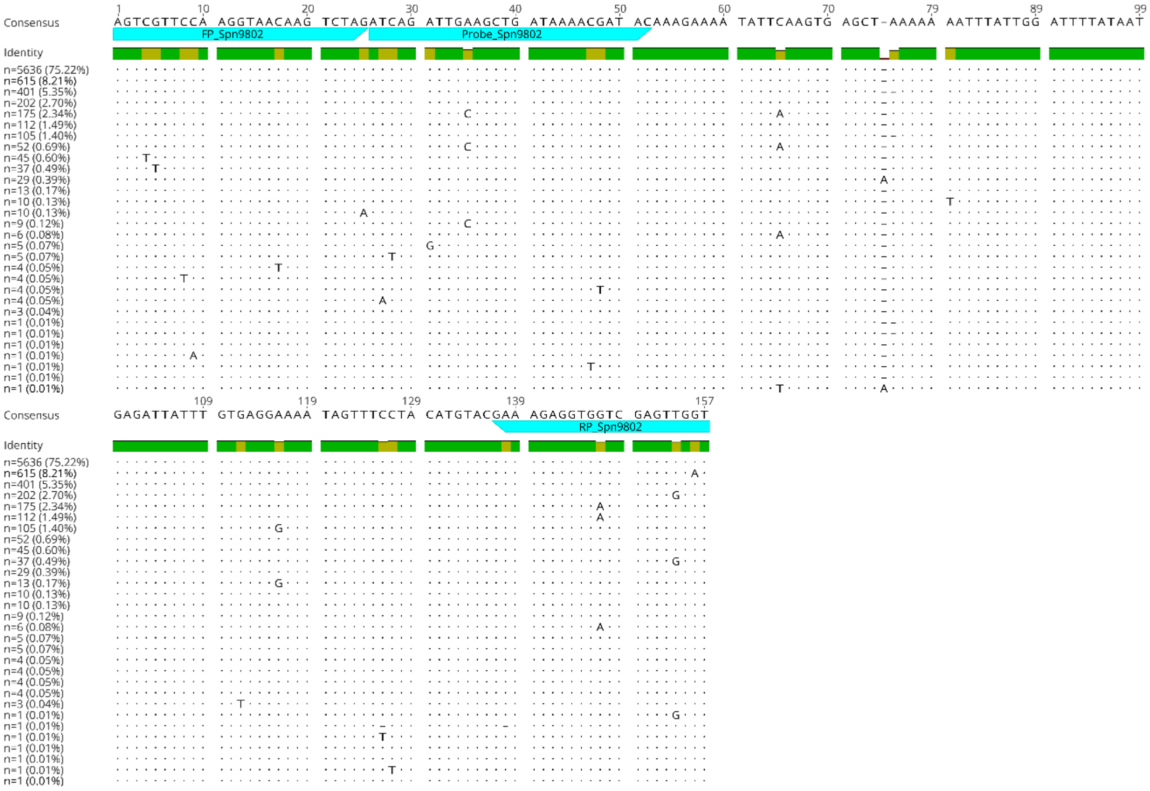

Supplementary Figure 4. Nucleotide sequence alignments of *in silico* PCR amplicons for two previously published targets. The consensus sequence was determined based upon the nucleotide in each position that represents at least 50% of nucleotides in that position, across the set of sequences being compared. Primer and probe binding regions are annotated (FP, forward primer; RP, reverse primer). The 'identity' row depicts nucleotide sequence pairwise identity at each position: green, 100%; brown, 30-100%. In the first column, n (%) indicates the frequency of that amplicon sequence out of the total amount of amplicons found in the *in silico* pneumococcal study dataset for that assay. Dots indicate that the nucleotide is identical to the consensus; dashes indicate a sequence gap.

Unique SP2020_new *in silico* amplicons

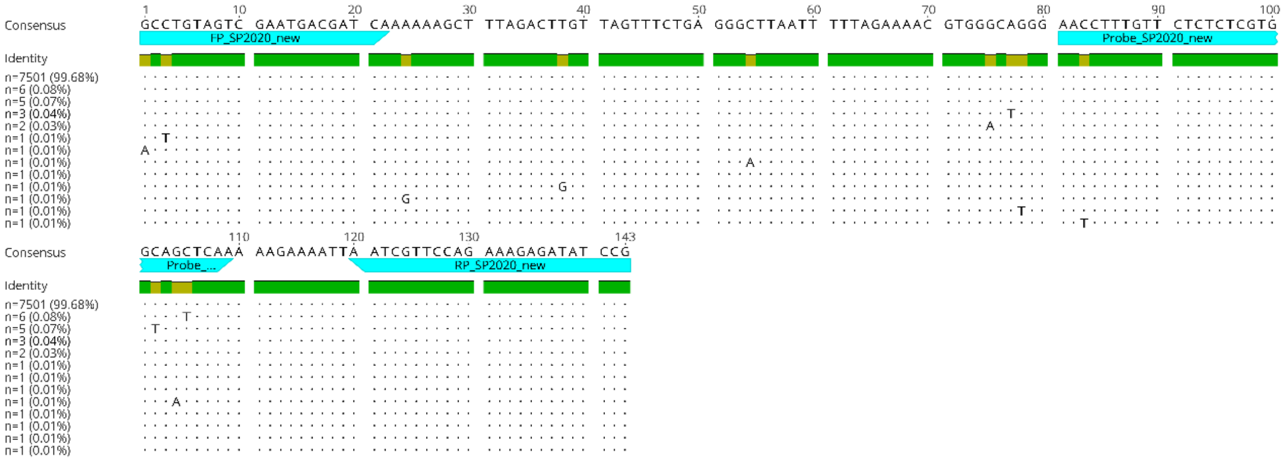

Unique Xisco_1 *in silico* amplicons

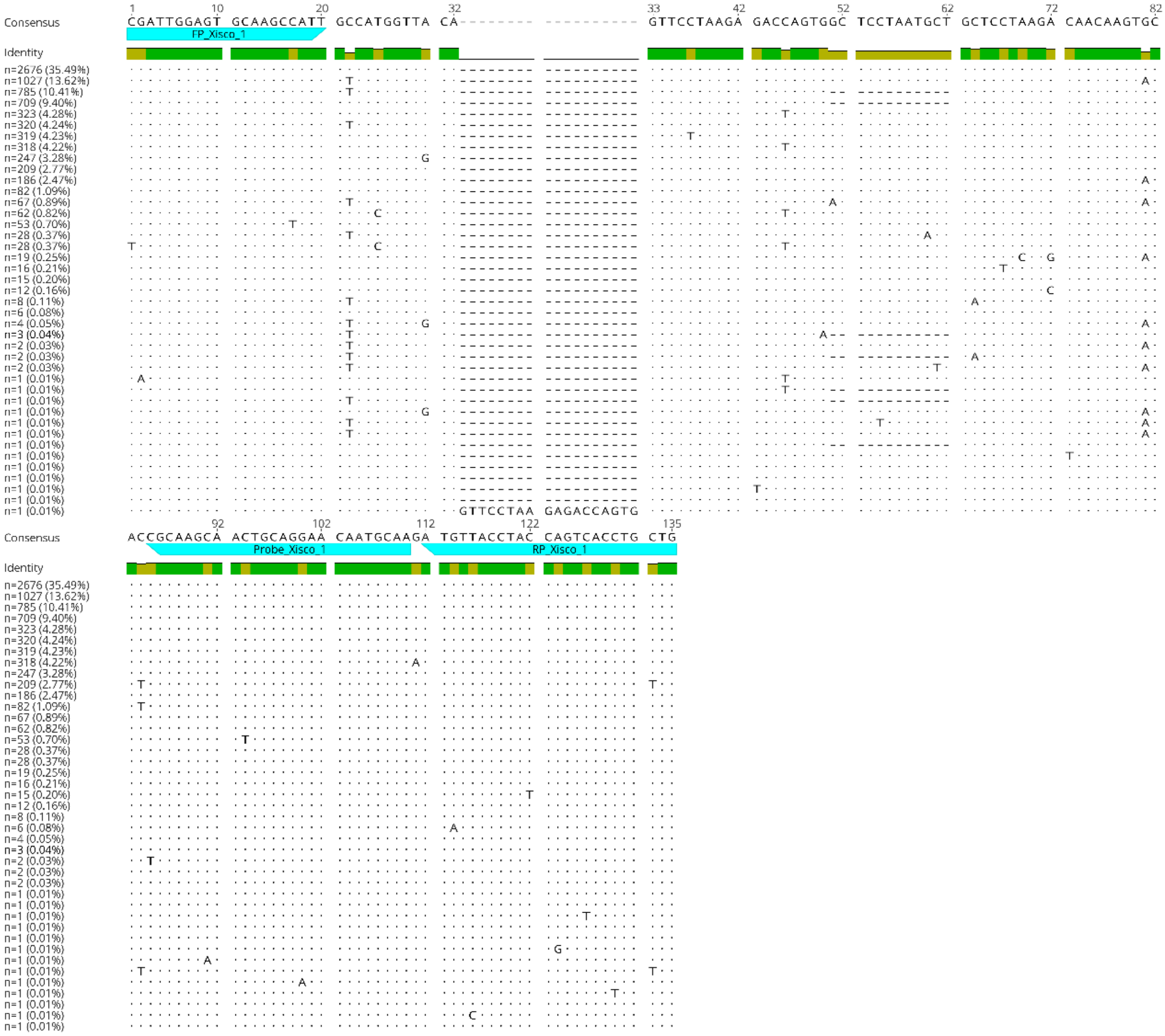

Supplementary Figure 5. Nucleotide sequence alignments of *in silico* PCR amplicons for two new assays. The consensus sequence was determined based upon the nucleotide in each position that represents at least 50% of nucleotides in that position, across the set of sequences being compared. Primer and probe binding regions

are annotated (FP, forward primer; RP, reverse primer). The 'identity' row depicts nucleotide sequence pairwise identity at each position: green, 100%; brown, 30-100%. In the first column, n (%) indicates the frequency of that amplicon sequence out of the total amount of amplicons found in the *in silico* pneumococcal study dataset for that assay. Dots indicate that the nucleotide is identical to the consensus; dashes indicate a sequence gap.

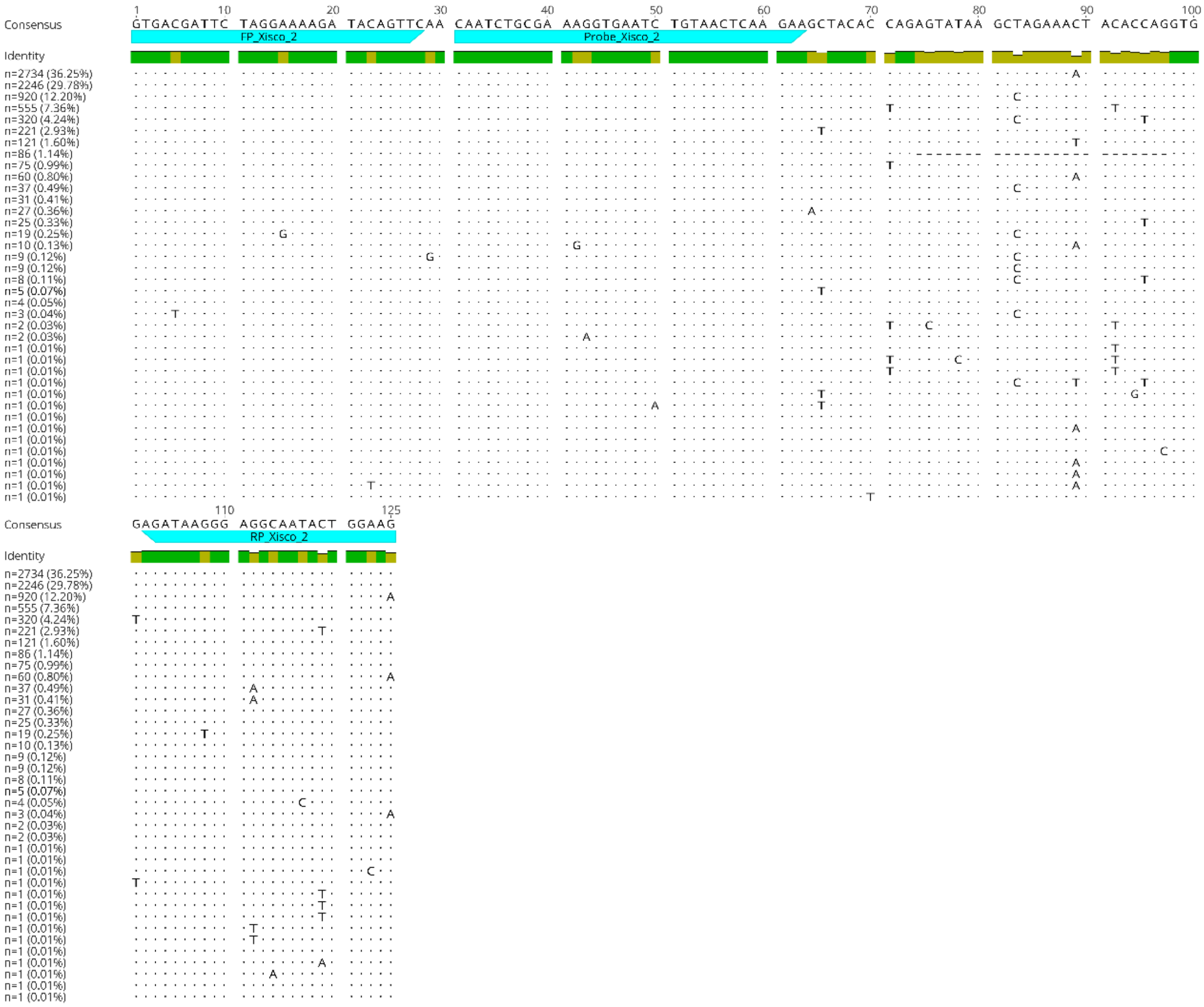

Supplementary Figure 6. Nucleotide sequence alignments of unique Xisco_2 *in silico* amplicons. The consensus sequence was determined based upon the nucleotide in each position that represents at least 50% of nucleotides in that position, across the set of sequences being compared. Primer and probe binding regions are annotated (FP, forward primer; RP, reverse primer). The 'identity' row depicts nucleotide sequence pairwise identity at each position: green, 100%; brown, 30-100%. In the first column, n (%) indicates the frequency of that amplicon sequence out of the total amount of amplicons found in the *in silico* pneumococcal study dataset for that assay. Dots indicate that the nucleotide is identical to the consensus; dashes indicate a sequence gap.

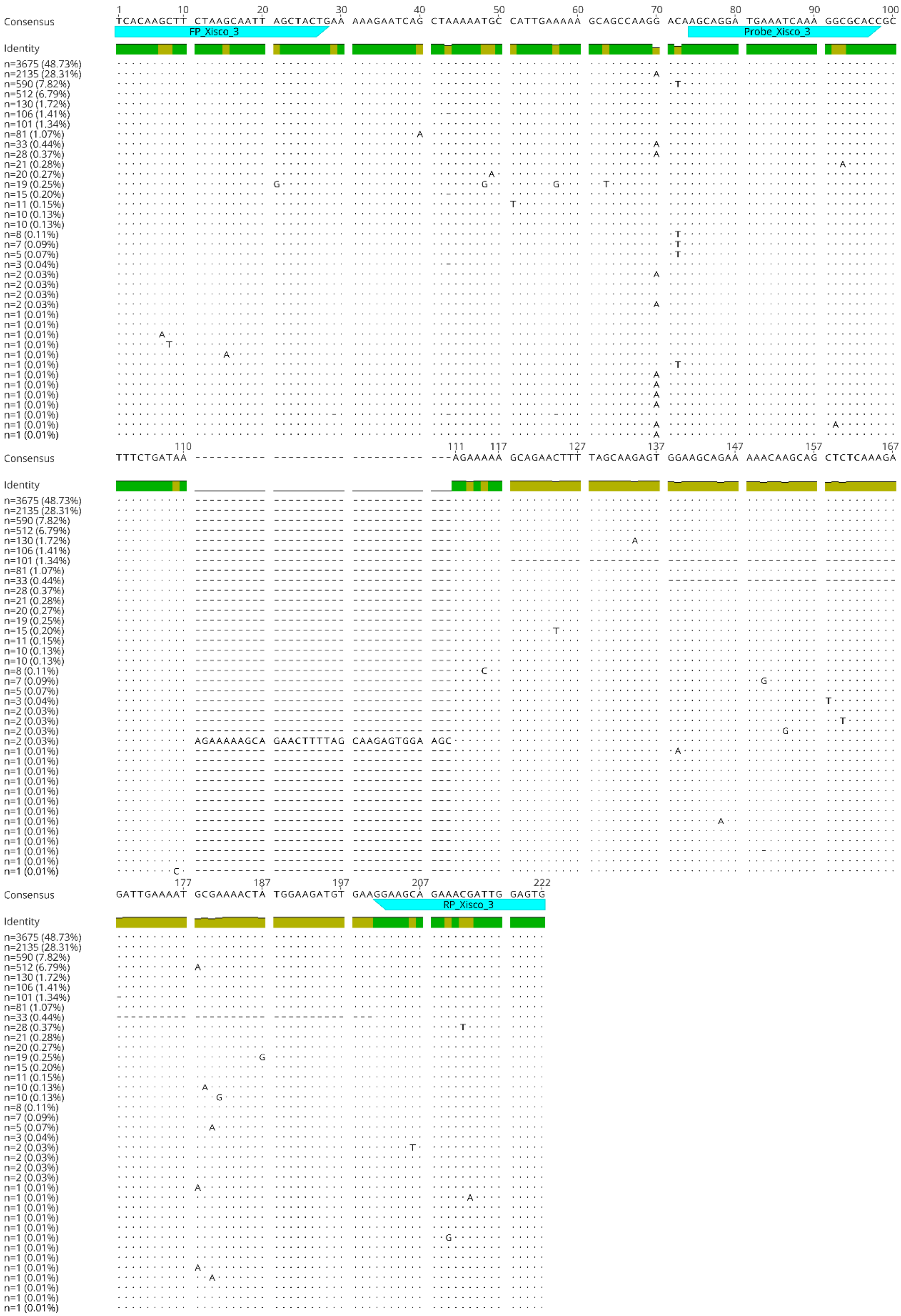
Consensus
TCACAAGCTT CTAAGCAATT AGCTACTGAA AAAGAATCAG CTAAAAATGC CATTGAAAAA GCAGCCAAGG ACAAGCAGGA TGAAATCAAA GGCGCACCGC
FP_Xisco_3
Probe_Xisco_3
Identity
n=3675 (48.73%)
n=2135 (28.31%)
n=590 (7.82%)
n=512 (6.79%)
n=130 (1.72%)
n=106 (1.41%)
n=101 (1.34%)
n=81 (1.07%)
n=33 (0.44%)
n=28 (0.37%)
n=21 (0.28%)
n=20 (0.27%)
n=19 (0.25%)
n=15 (0.20%)
n=11 (0.15%)
n=10 (0.13%)
n=10 (0.13%)
n=8 (0.11%)
n=7 (0.09%)
n=5 (0.07%)
n=3 (0.04%)
n=2 (0.03%)
n=2 (0.03%)
n=2 (0.03%)
n=2 (0.03%)
n=1 (0.01%)
Consensus
TTTCTGATAA ---------- ---------- ---------- ---AGAAAAA GCAGAACTTT TAGCAAGAGT GGAAGCAGAA AAACAAGCAG CTCTCAAAGA
AGAAAAAGCA GAACTTTTAG CAAGAGTGGA AGC
Consensus
GATTGAAAAT GCGAAAACTA TGGAAGATGT GAAGGAAGCA GAAACGATTG GAGTG
RP_Xisco_3

Supplementary Figure 7. Nucleotide sequence alignments of unique Xisco_3 *in silico* amplicons. The consensus sequence was determined based upon the nucleotide in each position that represents at least 50% of nucleotides in that position, across the set of sequences being compared. Primer and probe binding regions are annotated (FP, forward primer; RP, reverse primer). The 'identity' row depicts nucleotide sequence pairwise identity at each position: green, 100%; brown, 30-100%. In the first column, n (%) indicates the frequency of that amplicon sequence out of the total amount of amplicons found in the *in silico* pneumococcal study dataset for that assay. Dots indicate that the nucleotide is identical to the consensus; dashes indicate a sequence gap.

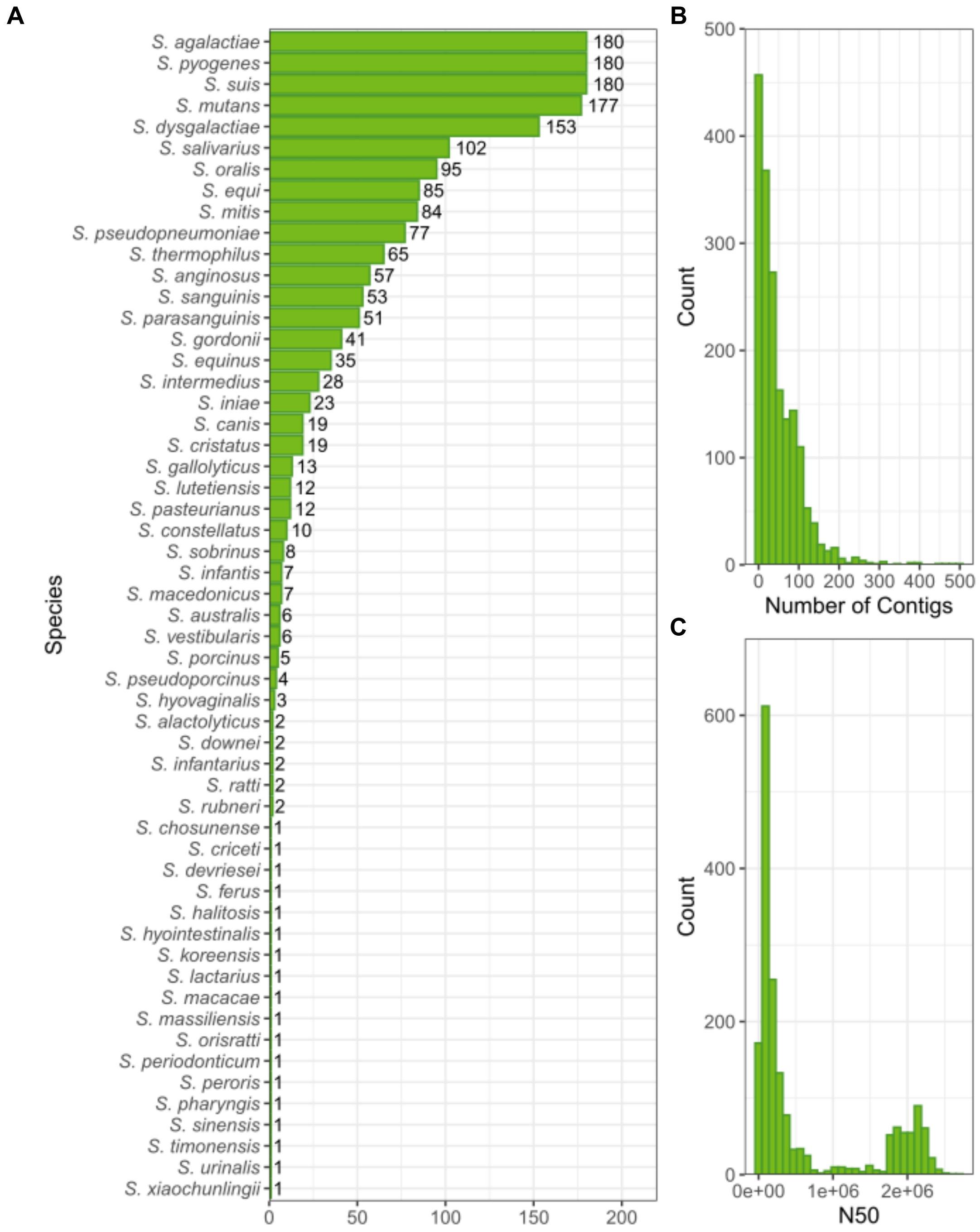

Supplementary Figure 8. Description of the non-pneumococcal *Streptococcus* (NPS) species genome dataset (n=1,825). (A) Frequency of each of the 55 different bacterial species. (B) Distribution of the number of contigs per NPS genome. (C) Distribution of the N50 values of the genome dataset.
