# Supplementary material for: Utilizing large and diverse bacterial genome datasets to improve the detection and identification of Streptococcus pneumoniae via PCR-based diagnostics
Source: Microb Genom. 2025 Jun 9;11(6):001418. doi: 10.1099/mgen.0.001418 (PMC12149409; doi:10.1099/mgen.0.001418)
Supplement: Uncited Supplementary Material 2. [file mgen-11-01418-s002.pdf]

Supplementary Table 1. Published and newly designed primers and probes used in PCR and qPCR assays.

Target[a]	Primer/ probe	Nucleotide sequence (5' to 3')	Amplicon size (bp)[b]	Reference
lytA	Forward	ACGCAATCTAGCAGATGAAGCA	75	[8]
	Reverse	TCGTGCGTTTTAATTCCAGCT		
	Probe	TGCCGAAAACGCTTGATACAGGGAG		
piaB	Forward	CATTGGTGGCTTAGTAAGTGCAA	104	[10]
	Reverse	TACTAACACAAGTTCCTGATAAGGCAAGT		
	Probe	TGTAAGCGGAAAAGCAGGCCTTACCC		
psaA	Forward	GCCCTAATAAATTGGAGGATCTAATGA	114	[8]
	Reverse	GACCAGAAGTTGTATCTTTTTTTCCG		
	Probe	CTAGCACATGCTACAAGAATGATTGCAGAAAGAAA		
ply	Forward	GCTTATGGGCGCCAAGTCTA	78	[8]
	Reverse	CAAAGCTTCAAAAGCAGCCTCTA		
	Probe	CTCAAGTTGGAAACCACGAGTAAGAGTGATGAA		
Spn9802	Forward	AGTCGTTCCAAGGTAACAAGTCTAG	157	[11]
	Reverse	ACCAACTCGACCACCTCTTTC		
	Probe	ATCAGATTGAAGCTGATAAAACGATAC		
SP2020	Forward	TAAACAGTTTGCCTGTAGTCG	155	[7]
	Reverse	CCCGGATATCTCTTTCTGGA		
	Probe	AACCTTTGTTCTCTCTCGTGGCAGCTCAA		
Xisco	Forward	TGACGATTCTAGGAAAAGATACAG	548	[13]
	Reverse	AGCAGGTGACTGGTAGGTAAC		
16S rDNA	Forward	TGTCGTCAGCTCGTGTCGTG	N/A	[27]
	Reverse	ACGTCATCCCCACCTTCCTC		
	Probe	TCCCGCAACGAGCGCAACCCTT		
SP2020_new	Forward	GCCTGTAGTCGAATGACGATCA	143	This study
	Reverse	CGGATATCTCTTTCTGGAACGATT		
	Probe[c]	AACCTTTGTTCTCTCTCGTGGCAGCTCAA		
Xisco_1	Forward	CGATTGGAGTGCAAGCCATT	135	This study
	Reverse	CAGCAGGTGACTGGTAGGTAACAT		
	Probe	TTGCATTGTTCCTGCAGTTGCTTGCG		
Xisco_2	Forward	GTGACGATTCTAGGAAAAGATACAGTTC	125	This study
	Reverse	CTTCCAGTATTGCCTCCCTTATCT		
	Probe	CAATCTGCGAAAGGTGAATCTGTAACTCAAGAA		
Xisco_3	Forward	TCACAAGCTTCTAAGCAATTAGCTACTG	222	This study
	Reverse	CACTCCAATCGTTTCTGCTTCC		
	Probe	AGCAGGATGAAATCAAAGGCGCACC		

a. All gene targets were used in qPCR assays except for Xisco, which was a standard PCR assay.
b. Amplicon size based upon the gene target in pneumococcal reference genome TIGR4 (PubMLST ID 37765).
c. The probe used in the SP2020_new assay was unchanged from the original SP2020 probe [7].

Supplementary Table 2. Limit of detection experiment of multiplex qPCR assay that contained pneumococcal targets Xisco_1, SP2020_new, and universal target 16S rDNA as a positive control.

**DNA per reaction (g)**	**DNA copies per reaction (n)**	**Run 1**		**Run 2**		**Run 3**	
		**Xisco_1**	**SP2020_new**	**Xisco_1**	**SP2020_new**	**Xisco_1**	**SP2020_new**
2.00E-10	87,568	21.0	21.2	21.0	21.3	20.8	21.1
2.00E-11	8,757	24.4	24.5	24.5	24.7	24.2	24.6
2.00E-12	876	27.7	27.9	27.9	28.1	27.7	28.0
2.00E-13	88	31.3	31.2	31.3	31.4	30.9	31.3
2.00E-14	9	35.5	34.2	35.9	34.8	35.8	34.8
1.00E-14	4	neg	35.2	36.8	neg	neg	neg
5.00E-15	2	neg	neg	neg	neg	37.8	38.4
3.00E-15	1	neg	neg	neg	neg	neg	neg

Note: neg, negative result. The quantification cycles (C_q) of three independent serial dilutions are shown. The following fluorescence thresholds were used: 0.1 for Xisco_1 and 0.01 for SP2020_new.

Supplementary Table 3. Limit of detection experiment of multiplex qPCR assay that contained pneumococcal targets Xisco_2, SP2020_new, and universal target 16S rDNA as a positive control.

**DNA per reaction (g)**	**DNA copies per reaction (n)**	**Run 1**		**Run 2**		**Run 3**	
		**Xisco_2**	**SP2020_new**	**Xisco_2**	**SP2020_new**	**Xisco_2**	**SP2020_new**
2.00E-10	87,568	19.8	18.9	20.0	19.2	19.7	19.1
2.00E-11	8,757	22.8	21.4	23.4	22.6	23.3	22.6
2.00E-12	876	26.4	25.6	26.9	26.2	26.9	26.2
2.00E-13	88	29.9	29.4	29.7	29.4	30.2	29.5
2.00E-14	9	33.6	33.0	34.7	35.4	34.6	34.6
1.00E-14	4	34.6	36.6	35.0	neg	35.1	34.0
5.00E-15	2	37.1	36.2	36.1	38.0	36.9	35.5
3.00E-15	1	35.7	35.7	neg	neg	37.0	37.0

Note: neg, negative result. The quantification cycles (C_q) of three independent serial dilutions are shown. The following fluorescence thresholds were used: 0.1 for Xisco_2 and 0.01 for SP2020_new.

Supplementary Table 4. Limit of detection experiment of multiplex qPCR assay that contained pneumococcal targets Xisco_3, SP2020_new, and universal target 16S rDNA as a positive control.

DNA per reaction (g)	DNA copies per reaction (n)	Run 1		Run 2		Run 3	
		Xisco_3	SP2020_new	Xisco_3	SP2020_new	Xisco_3	SP2020_new
2.00E-10	87,568	20.6	21.1	20.9	21.4	21.0	21.4
2.00E-11	8,757	24.2	24.7	24.6	24.9	23.8	24.6
2.00E-12	876	27.5	28.0	28.0	28.2	27.7	28.0
2.00E-13	88	31.3	31.4	31.3	31.7	31.2	31.4
2.00E-14	9	34.4	34.7	34.4	34.7	35.0	35.5
1.00E-14	4	35.2	35.8	36.0	35.6	35.0	35.0
5.00E-15	2	37.7	neg	neg	36.2	36.0	neg
3.00E-15	1	neg	39.6	neg	neg	neg	neg

Note: neg, negative result. The quantification cycles (C_q) of three independent serial dilutions are shown. The following fluorescence thresholds were used: 0.1 for Xisco_3 and 0.01 for SP2020_new.

Supplementary Table 5. Limit of detection experiment of multiplex qPCR assay that contained pneumococcal targets *lytA, piaB*, and universal target 16S rDNA as a positive control.

DNA per reaction (g)	DNA copies per reaction (n)	Run 1		Run 2		Run 3	
		lytA	*piaB*	*lytA*	*piaB*	*lytA*	*piaB*
2.00E-10	87,568	20.5	21.7	20.4	21.7	20.6	21.6
2.00E-11	8,757	23.9	25.0	24.0	25.0	23.7	24.9
2.00E-12	876	27.4	28.3	27.3	28.3	27.3	28.4
2.00E-13	88	30.4	31.6	30.9	31.5	30.9	31.7
2.00E-14	9	34.5	35.1	34.5	36.3	34.1	35.3
1.00E-14	4	34.3	36.0	35.6	neg	35.4	37.2
5.00E-15	2	35.6	36.5	neg	36.1	36.0	36.0
3.00E-15	1	36.8	neg	37.6	neg	37.2	36.7

Note: neg, negative result. The quantification cycles (C_q) of three independent serial dilutions are shown. The following fluorescence thresholds were used: 0.1 for *lytA* and 0.01 for *piaB*.

Supplementary Table 6. Results of the *in silico* analyses of pneumococcal genomes from UKHSA (n=6,090).

Target	Genomes with x amplicons (n)				Unique amplicons (n)	Range of amplicon length (bp)	Predicted amplicon positive (%) [$CI_{95\%}$]
	x=0	x=1	x=2	x=3			
lytA	71	5,544	448	27	11	75	98.8 (98.5-99.1)
piaB	84	6,006	0	0	15	104	98.6 (98.3-98.9)
SP2020_new	12	6,078	0	0	9	143	99.8 (99.7-99.9)
Xisco_1	0	6,090	0	0	45	105-165	100 (99.9-100)
Xisco_2	0	6,090	0	0	31	101-125	100 (99.9-100)
Xisco_3	1	6,089	0	0	44	159-222	100 (99.9-100)
